# SCHC over LoRaWAN Efficiency: Evaluation and Experimental Performance of Packet Fragmentation

**DOI:** 10.3390/s22041531

**Published:** 2022-02-16

**Authors:** Rodrigo Muñoz, Juan Saez Hidalgo, Felipe Canales, Diego Dujovne, Sandra Céspedes

**Affiliations:** 1 Department of Electrical Engineering, Universidad de Chile, Santiago 8370451, Chile; rmunozlara@ing.uchile.cl; 2 NIC Chile Research Labs, Universidad de Chile, Santiago 8320000, Chile; juan.saez.h@ing.uchile.cl (J.S.H.); felipe.canales.c@ing.uchile.cl (F.C.); 3 Faculty of Engineering, Universidad Diego Portales, Santiago 8370191, Chile; diego.dujovne@mail.udp.cl; 4 Department of Computer Science and Software Engineering, Concordia University, Montreal, QC H3G 1M8, Canada

**Keywords:** fragmentation, IPv6, LoRaWAN, LPWAN, SCHC

## Abstract

Low Power Wide Area Networks (LPWAN) are expected to enable the massive connectivity of small and constrained devices to the Internet of Things. Due to the restricted nature of both end devices and network links, LPWAN technologies employ network stacks where there is no interoperable network layer as a general case; instead, application data are usually placed directly into technology-specific two-layer frames. Besides not being able to run standard IP-based protocols at the end device, the lack of an IP layer also causes LPWAN segments to operate in an isolated manner, requiring middleboxes to interface non-IP LPWAN technologies with the IP world. The IETF has standardized a compression and fragmentation scheme, called Static Context Header Compression and Fragmentation (SCHC), which can compress and fragment IPv6 and UDP headers for LPWAN in a way that enables IP-based communications on the constrained end device. This article presents a model to determine the channel occupation efficiency based on the transmission times of SCHC messages in the upstream channel of a LoRaWAN™ link using the ACK-on-Error mode of standard SCHC. The model is compared against experimental data obtained from the transmission of packets that are fragmented using a SCHC over LoRaWAN implementation. This modeling provides a relationship between the channel occupancy efficiency, the spreading factor of LoRa™, and the probability of an error of a SCHC message. The results show that the model correctly predicts the efficiency in channel occupation for all spreading factors. Furthermore, the SCHC ACK-on-Error mode implementation for the upstream channel has been made fully available for further use by the research community.

## 1. Introduction

Nowadays, several channel access technologies serve the Internet of Things (IoT). Among them, LoRaWAN, SigFox, and NB-IoT have emerged as technologies designed to deliver high coverage while maintaining a low power consumption. These characteristics are highly valued in IoT because they can support a high density of end devices where periodic battery changes are not feasible or where the available power is limited.

Low Power Wide Area Network (LPWAN) technologies do not support native IP addressing because the reduced storage space cannot store a full protocol stack or fragment messages at the link layer. As a consequence, it is not possible to connect the end nodes directly to the Internet. Moreover, the lack of IP support prevents the use of standard management protocols and direct access to end devices. One example of the aforementioned problem is the Long Range Wide Area Networks, or LoRaWAN specification. LoRaWAN belongs to the LPWAN. LoRaWAN has a Maximum Transmission Unit (MTU) in the order of tens of bytes—without link-layer fragmentation—limiting the transmission of standard Internet upper-layer protocols, such as Internet Protocol (IP), Transmission Control Protocol (TCP), User Datagram Protocol (UDP), Hypertext Transfer Protocol (HTTP), and Constrained Application Protocol (CoAP). Furthermore, in the LoRaWAN architecture, each end node cannot be addressed directly from the Internet. Instead, a central module called a network server receives the messages destined for the end nodes and translates between a Uniform Resource Locator (URL) and an identifier of the LoRaWAN end device.

The Internet Engineering Task Force (IETF) has developed a specification, the Static Context Header Compression and Fragmentation (SCHC) [1], that enables header compression for IPv6, UDP, and CoAP protocols for LPWAN technologies. This compression and fragmentation permit communication through the Internet with an end device directly to the assigned IP address. The LPWAN technology used in this work is LoRaWAN. The specification that defines the operation of SCHC over LoRaWAN is RFC9011 [2].

The main contribution of this work is to create a model to determine the channel occupancy efficiency of the ACK-on-Error fragmentation mode of the SCHC specification. The model provides information for decision-making regarding which spreading factor to choose based on the efficiency of the channel. The model is based on the transmission periods that occur when the protocol running on top of LoRaWAN (e.g., IP) sends a large message that does not fit in a LoRaWAN packet (i.e., it is larger than the MTU of the access technology). The situation forces the use of the fragmentation mechanism provided by the SCHC standard [1]. We evaluate the transmission periods of a SCHC Fragment when values are modified in the fragmentation sublayer. Besides the model, we provide both experimental and theoretical evaluations of the transmission periods in the upstream channel of a LoRaWAN link, using the ACK-on-Error fragmentation mode from the SCHC specification. This model shows how the channel occupancy efficiency, latency, and spreading factor are inter-related when using SCHC in LoRaWan: information that allows the end user to make key decisions about which spreading factor to select.

The remainder of the article is organized as follows: In Section 3, we provide an overview of the SCHC framework and the SCHC over LoRaWAN. Section 4 focuses on the mathematical model proposed to analyze the ACK-on-Error mode channel efficiency performance. Section 5 describes the testbed employed for the experiments and Section 6 discusses the theoretical and experimental evaluations. Section 7 explains how our results can be applied to state-of-the-art LPWAN technologies such as LoRaWAN. Finally, we conclude the paper in Section 8.

## 2. Related Works

In this section, we describe the works related to the modeling of the performance of the SCHC standard. Since the standard was finalized recently—SCHC in 2020 and SCHC over LoRaWAN in 2021—there is still uncertainty regarding its efficiency and performance under different LPWAN conditions. We surveyed previous works that propose models evaluating different aspects of the performance of SCHC when used over Sigfox and LoRaWAN technologies. In [3], the author chose several performance metrics for an experimental evaluation of SCHC over Sigfox. The analysis focuses on metrics such as the transmission duration of a packet (which is evaluated both theoretically and experimentally), the amount of uplink and downlink messages exchanged to transmit a SCHC Packet, and the energy performance. In [4], Aguilar et al. propose the modeling and evaluation of packet transfer times, and the required number of uplink and downlink messages in a Sigfox network, considering the conditions when the end device is deployed in different radio zones, i.e., with different physical transmission rates and duty cycles. Sanchez-Gomez et al. [5], provide an experimental evaluation of SCHC over LoRaWAN to determine the latency and delivery ratio improvements as a consequence of different compression and fragmentation levels. In addition, the authors also examine the relationship between the overhead and useful payload sent per fragment. Aguilar et al. [6] present a model to evaluate the performance of the SCHC ACK-on-Error mode, applicable to the generic standard; hence, it may be considered for both Sigfox and LoRaWAN technologies. The model considers the number of SCHC ACK messages on the downlink to evaluate four metrics, namely the ACK message overhead, ACK bit overhead, ACK bit overhead with L2 headers, and the percentage of used bits per fragment. The theoretical results show that the channel occupancy has a direct relationship with the parameters of the SCHC specification: the tile and window sizes. In [7], the authors perform an analysis of the fragmentation modes of the SCHC specification [1]. The work introduces three performance metrics: channel occupancy (CO), goodput, and the total delay. Regarding the CO metric, the authors do not describe how CO is calculated. They also indicate that the channel occupancy was calculated without considering errors in the message reception. Our model does consider these errors. The authors simulated an error-free scenario using the OpenSCHC [8] simulation tool. In the no-ACK mode, the results were shown to have the lowest total delay and the highest goodput. In addition, the results showed that the total delay and channel occupancy, in both ACK-always and ACK-on-Error modes, are directly proportional to the number of windows required to transmit a SCHC Packet.

Table 1 is a list summarizing the related works. The table shows a reference to the publications and the technology used in the model (LoRaWAN or Sigfox), in addition to the metrics used. The works presented in this section contribute to determining the performance of SCHC. Nevertheless, previous works did not address the channel efficiency analysis in the presence of errors, which is critical in constrained networks and devices such as the ones employed with LoRaWAN. Therefore, in this work, we design a new model to determine the channel efficiency that is evaluated both theoretically and experimentally using a real LoRaWAN implementation.

## 3. SCHC over LoRaWAN

This section describes the layer model of the SCHC standard and explains the concept of fragmentation used in SCHC. We review the modes of operation necessary to fragment a packet and show the types of SCHC messages to transport the original packet fragments. Finally, we explain how SCHC fragmentation works when operating over LoRaWAN.

### 3.1. Brief Introduction to SCHC

**Figure 1:**
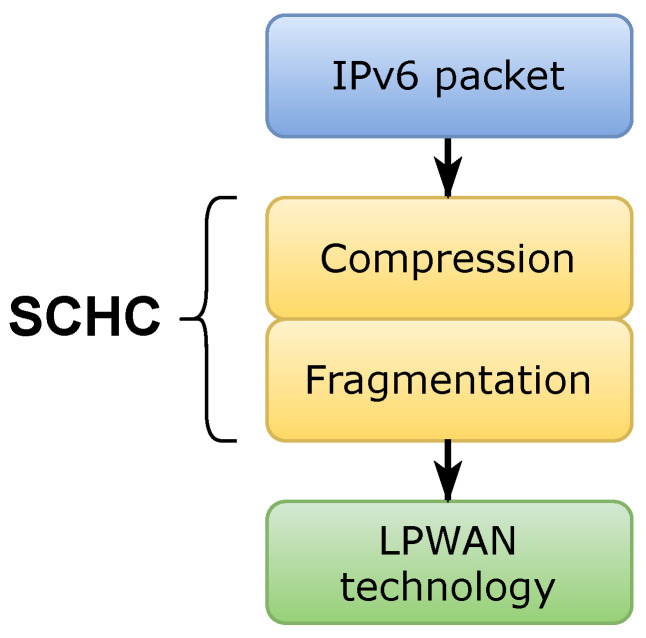
Location of the compression and fragmentation sublayers for the SCHC standard.

The SCHC specification proposal [1], henceforth SCHC, establishes the compression and fragmentation functionalities of the low power wide area networks family technologies: Sigfox, LoRaWAN™, and NB-IoT. SCHC is considered an adaptation layer between a network layer protocol such as IPv6 and a data link layer such as LPWAN-type technologies. This adaptation layer is made up of two sublayers called compression and fragmentation, as shown in Figure 1.

The Maximum Transmission Unit (MTU) is the maximum data size that a protocol can transport as useful information. On the other hand, the spreading factor reflects the relationship between the symbol rate and the chirp rate. A lower spreading factor decreases the sensitivity and range, but also shortens the airtime of a packet and decreases the probability of a collision. LoRaWAN uses six different spreading factors numbered 7 to 12. The higher the spreading factor, the lower the MTUs.

**Figure 2:**
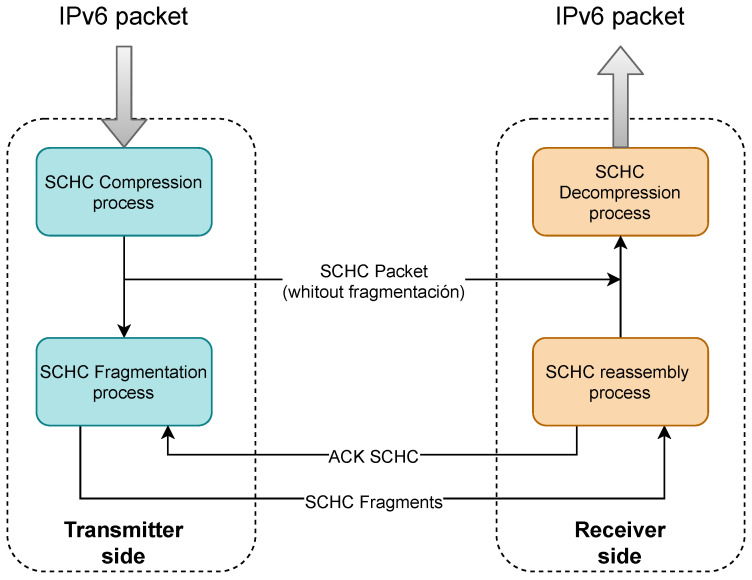
Flow that a message follows when entering the SCHC stack both in the transmitter and in the receiver of the message. Note that the message can go through only the compression layer or through both layers (compression/fragmentation).

Before an IPv6 packet is transmitted, it is processed by the SCHC compression sublayer. The result of this process is a SCHC Packet. If the SCHC Packet is less than or equal to the MTU of the LPWAN protocol, it is transmitted without fragmentation; otherwise, the SCHC Packet is delivered to the fragmentation sublayer. Figure 2 illustrates the transmission process of an IPv6 packet as it passes through the SCHC sublayers. The fragmentation sublayer and its modes of operation are explained below as a theoretical basis for understanding the study of efficiency in this sublayer.

### 3.2. SCHC Fragmentation Concepts

**Figure 3:**
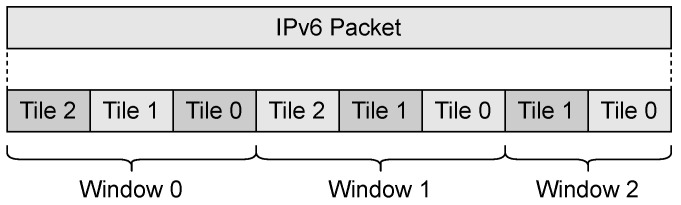
Representation of a SCHC Packet being split into tiles and windows within the fragmentation sublayer.

In LPWAN technologies, the Maximum Link-Layer Transmission Unit or MTU is of the order of 100s bytes. The small MTU size coupled with the lack of a fragmentation mechanism prevents the transport of protocols based on the Internet protocol, but the SCHC specification includes a fragmentation mechanism that allows for the transport of IPv6 packets. It also defines elements of the fragmentation mechanism, including tiles, windows, messages, and fields. Tiles are the segments that SCHC Packets are fragmented into. A group of tiles compose a window. Figure 3 illustrates this concept.

In order to successfully transmit the tiles from the transmitter to the receiver, SCHC uses five types of messages:

- SCHC Fragment: message sent from the transmitter to the receiver. It transports several tiles. There are two types of messages in this category:
  – SCHC Regular Fragments: are generally used to carry tiles that are not the last one of a SCHC Packet;
  – All-1 SCHC Fragments: are used to carry the last tile of the SCHC Packet;
- SCHC ACK: message sent from the receiver to the transmitter. It is used to confirm or not confirm the reception of one or more tiles;
- SCHC ACK REQ: message sent from the transmitter to the receiver requesting the confirmation of one or more tiles through a SCHC ACK;
- SCHC Sender-Abort: message sent from the transmitter to the receiver indicating that the transmitter has canceled the transmission of fragments;
- SCHC Receiver-Abort: message sent from the receiver to the transmitter indicating to cancel the transmission of fragments.

All messages listed above can contain one or more fields or headers. The fields or headers are the following:

- **Rule ID**: This field is present in all messages and is used to identify whether or not a message is a SCHC Fragment;
- **Datagram Tag (DTag)**: This field uniquely identifies two fragments that belong to two different SCHC Packets. It has a length of T bits;
- **W**: When the transmitted SCHC Fragments are grouped into windows, this field carries the number of the window to which the fragment belongs. It has a length of M bits;
- **Fragment Compressed Number (FCN)**: This field indicates the sequence number of the tiles. It has a length of N bits;
- **Reassembly Check Sequence (RCS)**: This field only appears in All-1 SCHC Fragment messages. It transports the error detection code based on the CRC32 polynomial. It has a length of U bits;
- **C (Integrity Check)**: This field is 1 bit in size and is used in the SCHC ACK message to report the integrity check of the reassembled SCHC Packet. A value of 1 indicates that the integrity check was performed and was successful. A value of 0 indicates that the integrity check was not performed or that it was an error;
- **Compressed Bitmap**: This field appears in the SCHC ACK message to report on the receiver bitmap. The bitmap indicates which tiles were received correctly and which were not.

**Figure 4:**
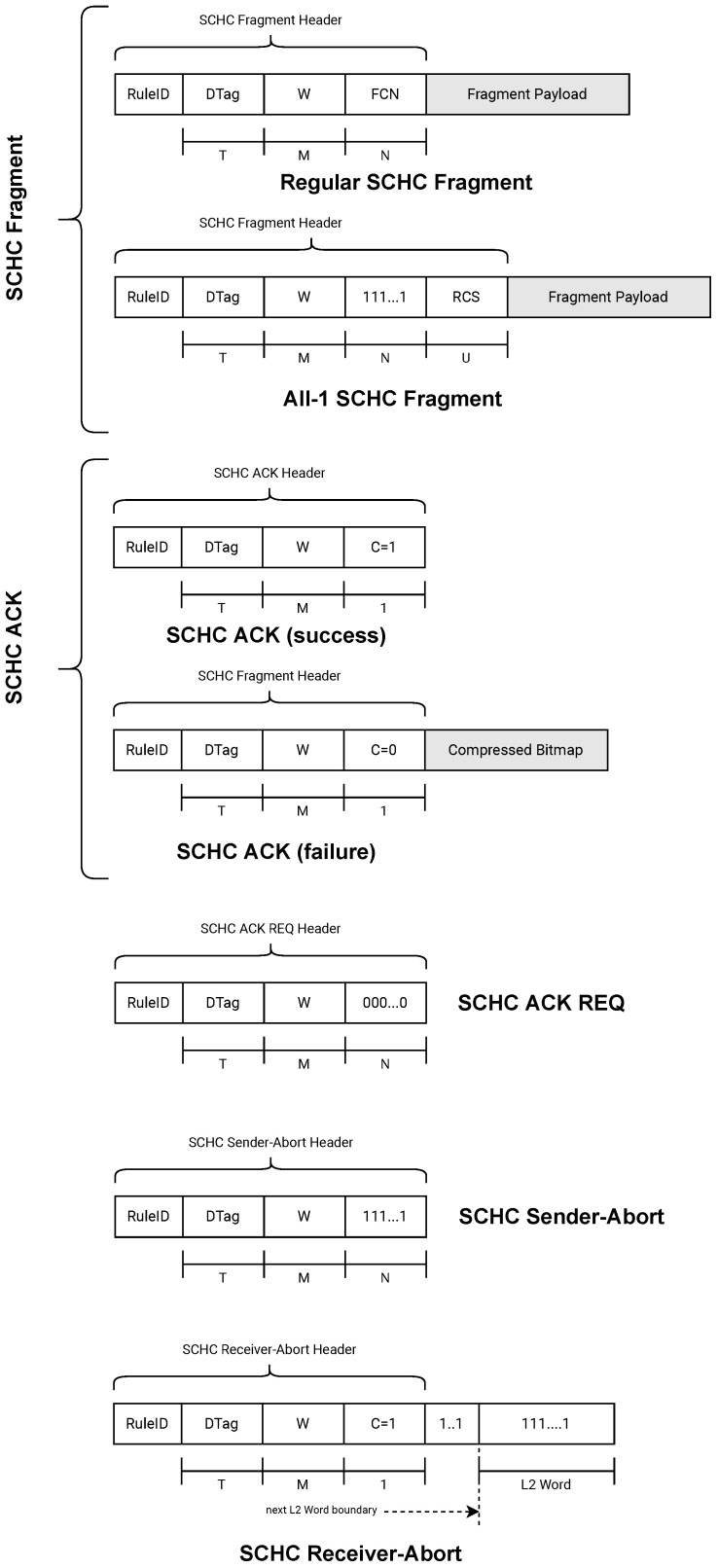
Five types of SCHC messages. The SCHC Fragment is subdivided into two subtypes of messages (Regular SCHC Fragment and All-1 SCHC Fragment).

Figure 4 shows the types of messages and headers used for the fragmentation and transmission of a SCHC Packet.

### 3.3. SCHC Fragmentation over LoRaWAN

The SCHC specification in RFC8724 [1] presents the modes of operation for the fragmentation of a SCHC Packet: No-ACK, ACK-Always, and ACK-on-Error. These modes define the general operation used in all Low-Power Wide Area Network (LPWAN) technologies defined in RFC8376 [9]. However, for efficient performance, some parameters and modes of operation need to be set appropriately for each LPWAN technology or profile. This section describes the parameters and modes of operation when SCHC is used over LoRaWAN networks based on specification RFC9011 [2]. The LoRaWAN protocol is specified by the LoRa Alliance in [10].

LoRaWAN uses only two of the three modes defined in [1]: ACK-on-Error and ACK-Always. The ACK-on-Error mode is used for uplink traffic and ACK-Always for downlink traffic. This work focuses on the fragmentation of the transmission of an IPv6 packet from the end device to the SCHC gateway. The ACK-on-Error fragmentation mode supports a variable MTU and the out-of-order delivery of fragments. Furthermore, SCHC fragmentation for LoRaWAN uses the concept of windows and tiles defined in Section 3.1 and shown graphically in Figure 3. All tiles must be of the same size, except for the last one. The size of the last tile must be smaller than or equal to the regular tile size.

Remember that an IPv6 packet is divided into several tiles, and that several tiles can be grouped in several windows, as shown in Figure 3. The number of tiles transported in a Regular SCHC Fragment depends on the MTU that exists at that moment. There is the possibility that a group of tiles belongs to the window *i* and another group of tiles to the window i+1, where all of them are transported in the same Regular SCHC Fragment. The last tile can be carried in a Regular SCHC Fragment or an All-1 SCHC Fragment.

## 4. Modeling Channel Occupation Using SCHC

### 4.1. Channel Occupancy Efficiency Estimate

In [11], the author presents the efficiency estimation for the ARQ protocols (see Chapter 5.2.4). This definition can be extended to other protocols using a clear and precise concept, such as the effective transmission rate.

According to [11], the efficiency $\eta_{0}$ is the **ratio** between the effective transmission rate $R_{eff}^{0}$ and the transmission rate of the channel named *R*, as indicated in the following equation:

(1) $$\begin{array}{c} \eta_{0}=\frac{R_{eff}^{0}}{R}=\frac{\frac{n_{bits}}{t_{0}}}{R} \end{array}$$

Thus, the effective transmission rate $R_{eff}^{0}$ is calculated as the **ratio** between the number of bits of information delivered to the recipient and the total time required to transmit that number of bits. Due to the possibility of transmission errors, $t_{0}$ in Equation (1) has a random behavior defined in Section 4.3. *R* is defined for each band in the document LoRaWAN™Regional Parameters [12].

For the case of the SCHC protocol using the ACK-on-Error mode, the number of bits of information delivered to the recipient is called $n_{bits}$ and is given by Equation (2):

(2) $$\begin{array}{c} n_{bits}=n_{tiles}\cdot tile_{size} \end{array}$$

If we represent this equation as a function of the number of SCHC Fragments, then the new expression will be Equation (3):

(3) $$\begin{array}{c} n_{bits}=n_{fragments}\cdot n_{tiles\_per\_fragments}\cdot tile_{size} \end{array}$$

where $n_{fragments}$ is the number of SCHC Fragments within a transmission window in ACK-on-Error mode, and $n_{tiles\_per\_fragments}$ is the number of tiles in a SCHC Fragment. In this model, we assume that all SCHC Fragments have the same size. $tile_{size}$ is the size of a tile in bits.

### 4.2. LoRa Frame Time Calculation

A frame is the protocol data unit (PDU) of layer two in the OSI model: a LoRa frame. Remember that the protocol layer structure is IPv6 over SCHC, SCHC over LoRaWAN, and LoRaWAN over LoRa. The frame time is the time it takes for the transmitter to send a frame over the wireless medium. The frame time calculation is based on the sum of the Time on Air (ToA) defined by Semtech [13] and the additional processing time $t_{proc}$ used by the end device (LoPy4, WisDuino, etc.) to send a LoRa message to the network. Equation (4) shows how the SCHC Fragment and SCHC ACK request times are calculated; both depend on the time on air and the processing time.

(4) $$\begin{array}{c} T_{F}=ToA_{SCHC\_fragment}+T_{frag\_proc}\\ T_{AR}=ToA_{SCHC\_ackreq}+T_{ackreq\_proc} \end{array}$$

When a user executes the send(msg) method, the program not only transmits the message, but also performs other tasks before returning. The code that describes these other tasks is established within the send method and cannot be externally changed. $t_{proc}$ time is calculated from the time used to execute the send method minus the time it takes to send in the wireless medium (ToA). $t_{proc}$ time is mainly an execution of other parts of the code necessary to send a frame in the wireless medium (e.g., build the frame to be sent).

The time on air bases its calculation, among other things, on the size of the payload carried in the message LoRaWAN™. Therefore, the frame time will be different if a Regular SCHC Fragment, an All-1 SCHC Fragment, or an SCHC ACK request is sent.

### 4.3. Theoretical Model

**Figure 5:**
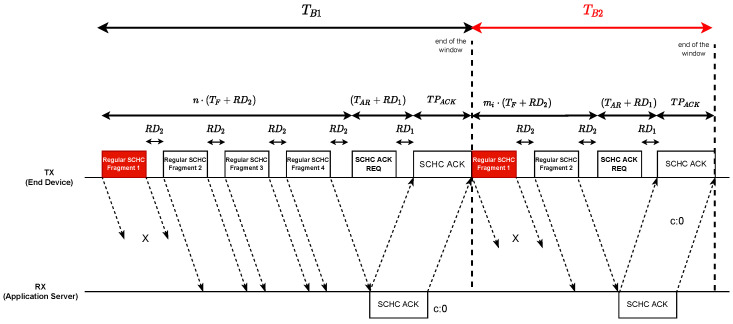
Message flow in ACK-on-Error mode [own elaboration].

The theoretical model describing SCHC’s fragmentation behavior is based on the following assumptions:

- The transmission of a SCHC Packet comprises a transmission window and *r* retransmission windows of the lost Regular SCHC Fragments;
- For the transmission of all of the SCHC Fragments belonging to a single IPv6 packet, the LoRa spreading factor is the same for all Regular SCHC Fragments;
- For simplicity, all Regular SCHC Fragments have the same number of tiles;
- All messages in the downlink are received by the end device in the first LoRa reception window, that is, at the end of the RECEIVE_DELAY_1 timer or $RD_{1}$ timer [12]. In this way, the node works in Class A, which is the most restrictive mode of operation;
- The processing of an All-1 SCHC Fragment at the receiver takes longer than the maximum length of the receive windows on the end device. For this reason the node waits for the next message in the uplink to open the window again and thus receive the SCHC ACK message sent in response to an All-1 SCHC Fragment (see Figure 5);
- Our implementation uses a Regular SCHC Fragment to transport the last tile. When one of the Regular SCHC Fragments is lost, the receiver must send a SCHC ACK message to the sender indicating which tiles arrived correctly and which did not. The SCHC ACK message only reports the receipt of tiles belonging to a window. The standard recommends that the SCHC receiver sends a SCHC ACK after every window, even if there is no missing tile. The SCHC sender waits for the SCHC ACK from the SCHC receiver before sending tiles from the next window. If the SCHC ACK is not received, the transmitter sends a SCHC ACK REQ message that can be retransmitted up to seven times.

The SCHC ACK message sent by the receiver requests the retransmission of the tiles that are missing and stores in the buffer the tiles that have been received correctly, with the aim to rebuild the message when missing tiles are retransmitted.

To illustrate this, the message flow shown in Figure 5 shows that it is possible to separate the transmission with fragment losses in two message blocks.

#### 4.3.1. Block 1

This block is made up of the complete sequence of the Regular SCHC Fragment messages that make up the first transmission window. The window ends when the SCHC ACK message has been processed on the sender side. The SCHC ACK message was sent by the receiver, indicating which tiles were received and which were not in the last window. This block has a duration time $T_{B1}$ given by Equation (5).

(5) $$\begin{array}{c} T_{B1}=n\cdot \left(T_{F}+RD_{2}\right)+\left(T_{AR}+RD_{1}\right)+TP_{ACK} \end{array}$$

where

- $T_{F}$ is the time it takes to transmit a Regular SCHC Fragment message over the wireless medium. Its calculation is explained in Section 4.2;
- *n* is the amount of Regular SCHC Fragments that must be sent to transmit all tiles within a transmission window. Remember that the model considers one transmission window and several retransmission windows;
- RD1 is the time that elapses between the end of a transmission on the uplink and the end of the RECEIVE_DELAY_1 timer defined in [12];
- RD2 is the time that elapses between the end of a transmission on the uplink and the end of the RX2 timer defined in [12]. It corresponds to the sum between RECEIVE_DELAY_2 and RX2;
- $T_{AR}$ is the time to transmit a SCHC ACK REQ. Its calculation is explained in Section 4.2;
- $TP_{ACK}$ is the processing time of the SCHC ACK Packet.

#### 4.3.2. Block 2

Block 2 is composed of all of the retransmission windows. There may be more than one retransmission window as fragments can be lost again.

The number of Regular SCHC Fragments retransmitted in a window depends exclusively on the fragments lost in the immediately preceding window and can be modeled as a binomial random variable *X*. The random variable *X* can take values in S=0,1,2,3,…,n. The probability function of *X* is given by Equation (6).

(6) P(xj)=kj·pj·1−pk−j

where *k* is the amount of Regular SCHC Fragments of the previous retransmission window. There are (k−j) SCHC Fragments received successfully and *j* Regular SCHC Fragments that need to be retransmitted. *p* is the probability of losing a Regular SCHC Fragment in the previous window. The calculation of this error probability is based on the bit error rate of each bit that makes up a Regular SCHC Fragment according to the equation defined by Aguilar in [6], which is

(7) $$\begin{array}{c} p=BER^{F} \end{array}$$

*F* is the fragment size in bytes of the L2 MTU of the underlying LPWAN technology, and BER is the bit error rate.

The mean or expected value of a binomial random value is given by Equation (8).

(8) E[X]=∑j=0kj·kj·pj·1−pk−jE[X]=k·p

Thus, the mean or expected value of Regular SCHC Fragments retransmitted in a window is given by Equation (9).

(9) E[Xwi]=mi=∑j=0m(i−1)xj·m(i−1)j·pj·1−pm(i−1)−jE[Xwi]=mi=mi−1·p

where $m_{i}$ is the expected value of Regular SCHC Fragments retransmitted in the *i*-th window and $m_{i-1}$ is the expected value of Regular SCHC Fragments retransmitted in the (*i*− 1)-th window. Table 2 shows the expected value of Regular SCHC Fragments for each retransmission window.

In Table 2, *n* is the number of Regular SCHC Fragments in the transmission windows. This variable is the same as $n_{fragments}$ in Equation 3. Then, taking into account the SCHC fragmentation model and the duration of each Regular SCHC Fragment indicated in Figure 5, the time $T_{i}$ of each retransmission window is given by Equation (10):

(10) $$\begin{array}{c} T_{i}=m_{i}\cdot \left(T_{F}+RD_{2}\right)+\left(T_{AR}+RD_{1}\right)+TP_{ACK} \end{array}$$

The time of Block 2 is given by the sum of all of the times of each retransmission window. Equation (11) shows the above idea.

(11) $$\begin{array}{cc} T_{B2} & =\sum_{i=0}^{r}T_{i}\\ T_{B2} & =\sum_{i=0}^{r}\left\{m_{i}\cdot \left(T_{F}+RD_{2}\right)+\left(T_{AR}+RD_{1}\right)+TP_{ACK}\right\} \end{array}$$

Thus, the $t_{0}$ time in Equation (1) is defined by the sum between Equations (5) and (11), as shown by Equation (12).

(12) $$\begin{array}{c} t_{0}=T_{B1}+T_{B2} \end{array}$$

## 5. Testbed

**Figure 6:**
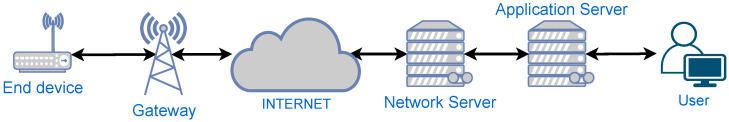
LPWAN architecture used in testbed.

To implement our model, we base our work on the diagram of a LPWAN defined in [1], which considers four blocks: end device, radio gateway, network server, and application server, as shown in Figure 6.

For the end device, we used a LoPy4 node. This node is a development board that supports four types of networks (LoRa, SigFox, WiFi, Bluetooth). The Firmware was developed in the built-in MicroPython intepreter, an efficient version of the Python3 programming language that includes a small subset of the Python standard library and is optimized to run on microcontrollers with limited resources, such as the ESP32 microcontroller.

The specifications of this development board are the following:

- CPU: Xtensa™dual – core 32 – bit LX6 microprocessor (s), up to 600 DMIPS;
- Memory: RAM: 520KB + 4MB and External flash: 8MB;
- WiFi: 802.11b/g/n 16mbps;
- RTC: Running at 150kHz;
- Security: SSL/TLS and WPA Enterprise security support;
- Hash/encryption: SHA, MD5, DES, AES.

For the radio gateway, we used a LoRaWAN ™gateway model RAK7240 from RAK Wireless [14]. The gateway is designed for outdoor use and comes with backhaul connectivity based on Wi-Fi, LTE, and Ethernet technologies, enabling multiple connectivity options to fall back on in the event of a network outage. The management and configuration firmware is based on OpenWRT. This gateway has dual LoRa concentrators for up to 16 channels, supports version 1.0.3 of the LoRaWAN ™stack, and is designed for the AU915 band ranging from 915 MHz to 928 MHz.

The network server we used is the The Things Network (TTN). The Things Network is a global network of gateways that deliver LoRaWAN coverage worldwide. The gateways are connected to the network servers of TTN from which the information sent by each sensor or end device can be obtained. The TTN’s backend handles message duplication, download message orchestration, and the management of platform integration.

A Linux server with a version of Ubuntu was used as the application server. This server has the Flask framework installed, which allows Python code PySCHC to be executed from an HTTP request. It should be noted that PySCHC is a Python implementation of the SCHC standard based on RFC8724 (SCHC: Generic Framework for Static Context Header Compression and Fragmentation) and RFC9011 (Static Context Header Compression and Fragmentation (SCHC) over LoRaWAN) and is implemented on both the end device side and the application server. The source code of the SCHC ACK-on-Error fragmentation mode is fully available in (https://github.com/niclabs/PySCHC) (accessed on: 26 December 2021).

## 6. Results

To estimate the channel occupancy efficiency, we employed the model proposed in Section 4.3. In particular, we used Equation (1). This equation depends on Equation (12), which models the transmission times of the SCHC messages defined in Figure 5. To determine the times of Equation (12), we used the values of Table 3, which shows the parameters applied to obtain the numerical results. In this table, the SCHC Fragment processing time and SCHC ACKReq processing time represent the additional time $t_{proc}$ used to send a LoRa message to the network, which is defined as a vector. The first element of the vector is the processing time for spreading factor 7; the second element corresponds to the spreading factor 8, and so on.

**Figure 7:**
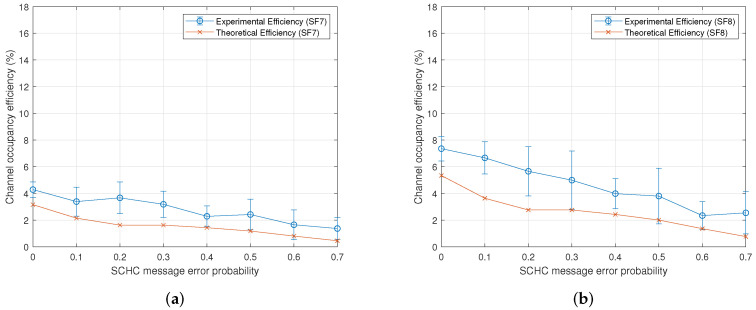

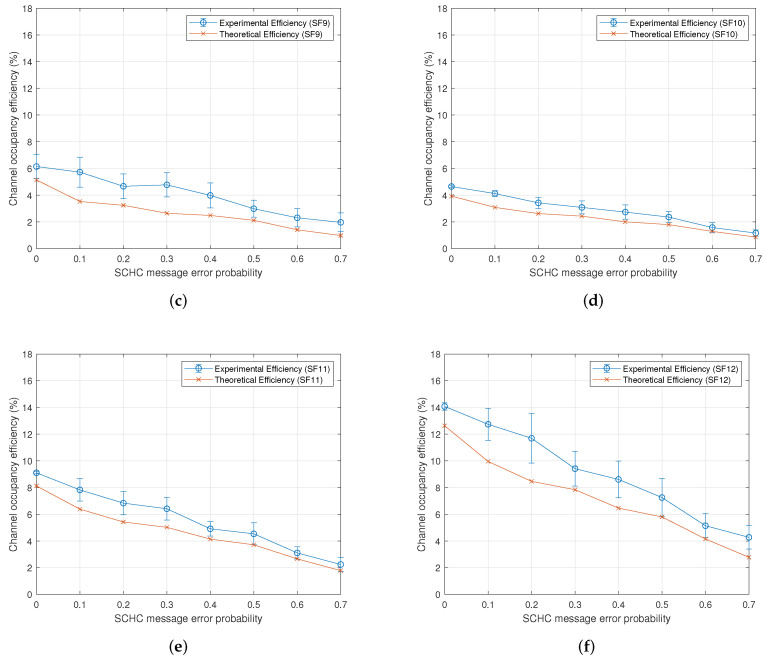
Experimental vs. theoretical channel occupancy efficiency for each spreading factor. (**a**) Spreading factor 7. (**b**) Spreading factor 8. (**c**) Spreading factor 9. (**d**) Spreading factor 10. (**e**) Spreading factor 11. (**f**) Spreading factor 12.

**Figure 8:**
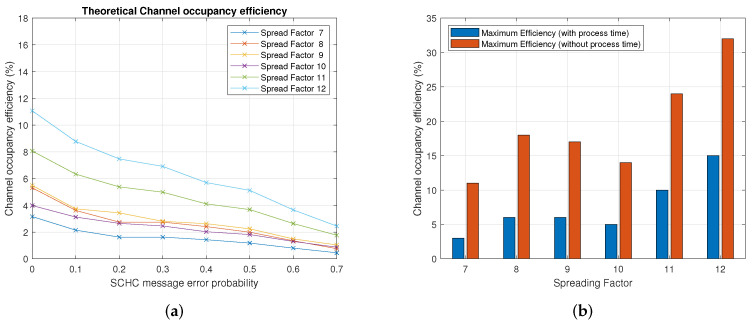
Channel occupancy efficiency. (**a**) Theoretical channel occupancy efficiency. (**b**) Maximum theoretical channel occupancy efficiency.

Figure 7 shows the channel occupancy efficiency for the eight data rates defined in Table 3. Each graph shows the experimental vs. theoretical efficiency for a particular spreading factor. Note that, for the theoretical efficiency, we have used Equation (1), and for the experimental efficiency, we employed the testbed described in Section 5. In general, the results show that the efficiency decreases when the probability of the loss of a SCHC message increases. Figure 8b shows the theoretical channel occupancy efficiency for each spreading factor. As can be seen, the spreading factors with the lowest and highest efficiency are the spreading factors 7 and 12, respectively. For any given probability, the efficiency increases when the spreading factor is increased. The above is valid except for spreading factor 10, for which the efficiency was observed to be between the spreading factors 7 and 8.

### 6.1. Maximizing Efficiency

The efficiency defined in (1) depends on three parameters: the physical bit rate R, the number of bits in a window, and the time to transmit those bits. The physical bit rate R is a value that depends on the LoRa data rate. For example, for data rate 0 (spreading factor 12), the R parameter in the AU915 band is 250 bit/seg. The number of bits in a SCHC window is 630 bytes (or 5040 bits). Thus, the only variable left to determine is the transmission time for those bits. Therefore, the highest efficiency is achieved when the transmission time is minimum, and this occurs when there are no errors or no loss of SCHC messages. Therefore, by looking at Figure 5, the transmission time of an error-free SCHC window is determined by (12) considering $T_{B2}=0$ because there are no errors in the SCHC messages.

The transmission time depends on how many regular SCHC Fragments (regular and all-1) need to be sent to transmit the 5040 bits of SCHC window. The number of SCHC Fragments depends on the size of the MTU, and the MTU depends on the spreading factor. Table 4 shows the number of tiles and the number of SCHC Fragment messages for a SCHC window for each data rate in the AU915 frequency band. For example, in data rate 0 for AU915, only 51-byte payloads are supported. Each SCHC message has a size of 50 bytes for the payload (five tiles of 10 bytes each) and 1 byte for the header.

To build 630 bytes of a SCHC window, we need 12 SCHC Fragment messages with its full payload (50 bytes of payload plus one byte of header), along with one SCHC Fragment message with its payload of three tiles (30 bytes of payload plus a header byte). This can be observed in the row with data rate 0 in Table 4. For example, consider Equation (1) to obtain the efficiency: to achieve a 100% efficiency when we transmit 5040 bits (630 bytes in a SCHC window) with a data rate 0 in the AU915 band, we need to transmit these data in 20.16 seconds (5040 bits at 250 bits per seconds of physical bit rate in spreading factor 12 or data rate 0).

### 6.2. Results with and without Processing Times

To analyze how the processing time affects the channel occupancy efficiency, we defined two cases: case 1 does not consider the processing time defined in (4). Case 2 considers the processing time. For case 1, the time involved in sending 13 SCHC Fragment messages plus receiving a SCHC ACK is given by Table 5.

In particular, for the case of data rate 0, the time of a SCHC window is 66970 [ms]. This time involves an efficiency of 32%. Table 6 summarizes the best-case efficiency for the data rates used in this work.

For case 2, Table 7 considers the same case as the previous paragraph, including the value of the processing time to calculate the channel occupancy efficiency. When processing times are included, the channel occupancy efficiency for data rate 0 decreases from 32% to 15%, showing the importance of the processing time on the channel occupancy efficiency. Figure 8b shows the maximum theoretical channel occupancy efficiency with and without the processing time for each spreading factor.

**Figure 9:**
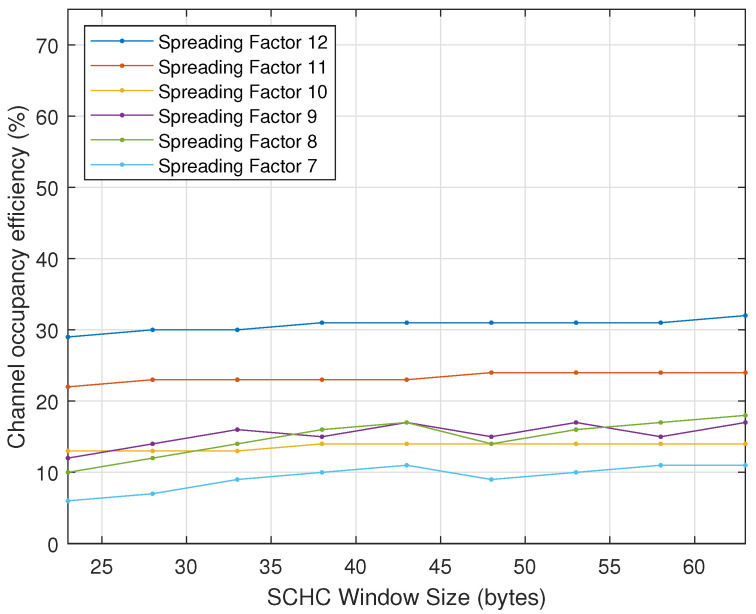
Maximum theoretical channel occupancy efficiency in function of SCHC window size and spreading factor.

Figure 9 shows the maximum theoretical channel occupancy efficiency as a function of the SCHC window size (in bytes) and the spreading factor, using a processing time equal to zero, without packet loss.

## 7. Discussion

The previous section’s results show the relationship between the channel occupancy efficiency and three communication parameters: the error probability of a SCHC message, the LoRa spreading factor, and the SCHC window size. Figure 7 shows an expected and intuitive behavior of the channel occupancy efficiency and error probability: there is a lower efficiency when the error probability of a SCHC message increases. The efficiency is calculated both experimentally and theoretically from the message transmission periods in a SCHC window using Equation (1). The message flow in Figure 5 is based on the SCHC standard defined in [1,2]. Using Equation (4), the only difference between the efficiency calculation using the model and the experimental data is related to the transmission periods of the SCHC Fragments and the SCHC ACK request.

The relationship between the channel occupancy efficiency and the spreading factor was not observed to be linear. The efficiency increases when the spreading factor increases, but an exception occurs for the spreading factor 10. This can be verified by looking at Table 6 and Table 7. In this table, we have extracted the maximum payload size and physical bit rates from [12]. The time on air was calculated from [13]. Thus, the maximum efficiency only depends on the frame duration, calculated from Table 5 and multiplied by the number of messages of each type, as defined in Table 4.

The final parameter studied was the SCHC window size. When focusing on the maximum theoretical channel occupancy efficiency, i.e., the efficiency when the error probability is zero, we can deduce that the maximum theoretical channel occupancy efficiency does not depend on the SCHC window size. This is interesting, as the IETF standard uses a window size of 10 bytes; however, our analysis showed that the maximum channel occupancy efficiency is constant regardless of the window size. Figure 9 shows this behaviour.

## 8. Conclusions

In this work, we have presented a model for determining the wireless channel usage efficiency for the SCHC standard on LoRaWAN technology. We showed that the model is accurate when compared to efficiency measurements from experimental results. Furthermore, we showed theoretically and experimentally that the efficiency decreases when the probability of error increases, and that this is true for all spreading factors. According to the results, there is no linear relationship between the spreading factor and efficiency since spreading factor 10 presents an atypical behavior resulting in an efficiency reduction compared to a hypothetical linear behavior. In summary, the efficiency is a consequence of the transmission period for each SCHC Fragment, which itself depends on the spreading factor and the error probability, whereas the window size does not apparently have a notable influence.

## 9. Future Works

This work investigated the upload channel using the ACK-on-Error mode of the SCHC protocol. Future work will apply the same approach to investigating the efficiency for the downlink using the ACK-Always mode over LoRaWAN™. 

## Figures and Tables

**Table 1:** List of related works that consider the modeling of the SCHC standard.

Publication	Technology	Type of Model	Metric
[3]	Sigfox	Theoretical Experimental	* Transmission duration of a packet * Uplink messages exchanged * Downlink messages exchanged * Energy performance
[4]	Sigfox	Theoretical Experimental	* Packet transfer times * Number of uplink messages * Number of downlink messages
[5]	LoRaWAN	Experimental	* Latency * Delivery ratio * Overhead in end node resources * Useful payload sent per fragment
[6]	Sigfox LoRaWAN	Theoretical	* ACK traffic * Quality of the radio link * SCHC F/R parameters
[7]	LoRaWAN	Simulation	* Total Channel Occupancy * Goodput of SCHC layer * Total Delay of SCHC layer

**Table 2:** Expected value of Regular SCHC Fragments for each retransmission window.

Retransmission Window	$m_{i}$
1st window	n·p
2nd window	$n\cdot p^{2}$
3rd window	$n\cdot p^{3}$
4th window	$n\cdot p^{4}$
:	:
rth window	$n\cdot p^{r}$

**Table 3:** Parameters used for the theoretical numerical results.

Parameter	Value
Data Rate	0, 1, 2, 3, 4, 5
LoRaWAN Frequency Band	AU915
SCHC Packet Size	892 bytes
SCHC Tile Size	10 bytes
SCHC Fragment Processing Time ($t_{frag\_proc}$)	DR5: 5223.6 [ms] DR4: 5300.8 [ms] DR3: 4661.7 [ms] DR2: 5101.6 [ms] DR1: 5285.2 [ms] DR0: 6634.2 [ms]
SCHC Ack Req Processing Time ($t_{ackreq\_proc}$)	DR5: 5053.7 [ms] DR4: 5053.7 [ms] DR3: 4661.7 [ms] DR2: 5101.6 [ms] DR1: 5285.2 [ms] DR0: 6634.2 [ms]

**Table 4:** n° of Tiles and n° of SCHC Fragment messages for a SCHC window (630 bytes) for each data rate in AU915 frequency band.

Data Rate	SF	Maximum Payload Size (Bytes)	Physical Bit Rate (Bps)	n° of Tiles in a SCHC Fragment (Full Payload)	n° of SCHC Fragment (Full Payload)	n° of Remaining Tiles
0	12	51	250	5	12	3
1	11	51	440	5	12	3
2	10	51	980	5	12	3
3	9	115	1760	11	5	8
4	8	222	3125	22	2	19
5	7	222	5470	22	2	19

**Table 5:** Time of frame for each SCHC message type (Fragment and ACK Req).

Data Rate	SF	SCHC Message Time		
		SCHC Fragment (Full Size) [ms]	SCHC Fragment (Not Full Size) [ms]	SCHC ACK REQ [ms]
0	12	9793.5	9138.1	7155.1
1	11	8560.6	8151	6659.5
2	10	7698.4	7534.5	6288.8
3	9	7656.4	7533.5	6164.9
4	8	7645.6	7574	6082.4
5	7	7368.9	7322.8	6046.3

**Table 6:** Maximum theoretical channel occupancy efficiency in AU915 band (without process time).

Data Rate	SF	Maximum Payload Size (Bytes)	Physical Bit Rate (Bps)	Total ToA (without Process Time) [ms]	Maximum Efficiency (without Process Time)
0	12	51	250	66,970	32%
1	11	51	440	50,197	24%
2	10	51	980	38,493	14%
3	9	115	1760	19,125	17%
4	8	222	3125	11,030	18%
5	7	222	5470	10,153	11%

**Table 7:** Maximum theoretical channel occupancy efficiency in AU915 band (with process time).

Data Rate	SF	Maximum Payload Size (Bytes)	Physical Bit Rate (Bps)	Total ToA (with Process Time) [ms]	Maximum Efficiency (with Process Time)
0	12	51	250	141,970	15%
1	11	51	440	125,197	10%
2	10	51	980	113,493	5%
3	9	115	1760	59,125	6%
4	8	222	3125	36,030	6%
5	7	222	5470	35,153	3%

## Data Availability

The datasets generated during and/or analyzed during the current study are available from the corresponding author on reasonable request.
